# The Consequences of Disdainful Hook-Ups for Later Egalitarian Relationships of Girls

**DOI:** 10.3390/ijerph18189521

**Published:** 2021-09-09

**Authors:** Ane López de Aguileta, Patricia Melgar, Elisabeth Torras-Gómez, Nerea Gutiérrez-Fernández

**Affiliations:** 1 Department of Sociology, University of Barcelona, Av. Diagonal 690, 08034 Barcelona, Spain; alopezdeaguileta@ub.edu; 2 Department of Education, University of Girona, Pl. Sant Domènec 9, 17004 Girona, Spain; patricia.melgar@udg.edu; 3 Faculty of Psychology and Education, University of Deusto, Av. De las Universidades 24, 48007 Bilbao, Spain; gutierreznerea@deusto.es

**Keywords:** intimate partner violence, masculinity, interpersonal relations

## Abstract

Introduction: There is extensive research about the consequences that toxic relationships with dominant masculinities have on different areas of women’s lives, including how they can influence future relationships they establish. Some of these women reproduce toxic relationships with dominant masculinities in their following relationships, and some women start to establish sexual-affective relationships with non-dominant masculinity models. However, less is known about the specific consequences in non-dominant masculinities who establish relationships with women who have not overcome the false memory of excitement regarding their toxic relationships. Methodology: In order to shed light on the consequences for non-dominant masculinities, eight communicative interviews were conducted with four women and four men. Results: The main findings indicate that those female participants who have kept an exciting memory of toxic relationships, when in an equal relationship, have attitudes of disdain towards non-dominant masculinities. They may ridicule them, not value their personal or intellectual abilities, talk down to them, and even negatively value their sexual aptitudes. Discussion: The application of the previous literature to our results leads to establishing the work on memories of relationships that have led to socialization in violent relationships as a preventive approach. Conclusion: The previously mentioned attitudes could generate health problems for non-dominant masculinities engaged in these relationships.

## 1. Introduction

The consequences gender violence has, in both stable and sporadic relationships, have been widely studied. Most studies have focused on the health consequences it has on women. Intimate partner violence (IPV hereinafter), such as physical assault and sexual coercion, is related to worse mental and physical health outcomes for women [1,2,3,4]. Depression, anxiety, suicidal thoughts or attempts, post-traumatic stress disorder and alcohol or drug abuse are among the most common mental health problems related to IPV [2]. Regarding physical health, violence against women is associated with injuries (including brain injuries), chronic health problems (allergies, breathing problems, among others), unwanted pregnancy, sexually transmitted infections, sexual dysfunction [2] and coronary heart disease [5]. In addition, it has been evidenced that these consequences can be prolonged over time, even long after the abusive relationship has ended [6]. 

As we can notice, the study of these consequences focuses on the health of the victims, but little has been studied on the social learning that this violence can entail. In his book *Pedagogy of the Oppressed*, Freire [7] explained the serious consequences of oppression. In this work, he detailed the path to liberation, but warned that the treatment inflicted by the oppressor can lead to an apprenticeship for the victims that leads them away from this path. In other words, those who are oppressed may end up harboring an oppressor within them as well. These ideas should be taken into account when analyzing IPV, since the relationship between victim and perpetrator corresponds to the relationship between oppressor and oppressed.

From a social learning perspective, the studies that have explored the consequences of IPV on future relationships have shed light on the essential role memory of past relationships and hook-ups has for later relationships [8,9,10]. Many girls who had previous violent hook-ups had told their friends a very different story of what had happened, omitting parts where violence was present by changing it into a language that showed excitement instead of the anxiety or disgust they felt in the moment, thus forgetting what really happened and storing a different memory of the situation [11]. Storing a false memory of a coerced relationship as exciting has been evidenced to increase the risk of victimization in future relationships [8,9]. In this way, research has shown that acceptance of IPV and having a history of violent dating are predictors of violence perpetration and victimization [12,13], and suffering from dating violence in adolescence might lead to developing a relationship pattern still persistent in adulthood [13]. We emphasize, therefore, that the consequences of a violent relationship not only affect the woman victim, but also affect non-violent men with whom they have relationships in the future.

The review of these past relationships is one of the key elements in the process of recovery from violence. Effective programs of intervention to overcome gender-based violence include reflection on previous violent relationships to prevent future victimization [14]. Women and girls who have been involved in programs focused on the reflection and memories of the socialization of violent affective-sexual relationships have been shown to increase critical memories of violent relationships and, thus, end toxic relationships or decide not to have such relationships in the future [8,9,10]. 

In summary, the violence cycle has been studied, which implies how a woman can stay trapped in a violent relationship [15,16]. It has also been researched how having violent relationships increases the risk of future IPV victimization [17]. However, what sequelae persist when subsequent relationships are no longer violent has not been explored.

Concretely, the consequences of not having overcome the false memory of past violent hook-ups or relationships in later relationships with non-dominant men are still underexplored. Thus, the aim of this study is to investigate the effects of maintaining an exciting memory of toxic relationships on relationships with non-dominant masculinities.

## 2. Materials and Methods

This research focused on the analysis of the consequences of gender-based violence for non-dominant masculinities—specifically, for non-dominant men who maintain relationships with women who have been victims of violence in the past and have not analyzed these relationships in depth and, therefore, have not transformed their memories in this respect. In order to analyze this reality, a qualitative study following the communicative approach [18] was undertaken. This methodological orientation is based on the assumption that knowledge is the result of dialogue between science and society. Concretely, communicative daily life stories have been collected, which is a cooperative process of understanding which is oriented towards transformation. Therefore, in the process of developing the story, researchers presented the main scientific results on the topic under study, and these ideas were analyzed and complemented by participants. In this way, they contributed their experiences and interpretations, contrasting them with scientific theories and research [19]. The resulting arguments presented in this article were the result of consensus.

In this article, theoretical perspectives on the preventive socialization of gender violence [20] and the pedagogy of the oppressed were taken as the reference [7,21]. When the term non-dominant men is used, it refers to those who do not correspond to attitudes of domination and abuse and, therefore, do not exercise gender-based violence. Following the terminology coined by Flecha, Puigvert and Rios [22], the category of non-dominant masculinity includes two different typologies: oppressed traditional masculinity and new alternative masculinities. It is important to note that only men who correspond to the new alternative masculinities contribute to overcoming gender-based violence. Men who respond to the traditional dominant and oppressed model of masculinity accept and reproduce the patriarchal system’s effects on women and also on “good” men. Oppressed masculinity accepts the established traditional system, sometimes actively and sometimes passively, as an accomplice of the traditional dominant masculinity and, at the same time, as a victim of it. He does not oppose violence against himself or others and, therefore, does not contribute to the construction of egalitarian relationships.

### 2.1. Participants

A total of 8 communicative daily life stories were conducted: 4 stories with women and 4 with men. All participants were over 18 years of age, and their participation was subject to the maintenance of anonymity and the use of the content of their stories solely for the purpose of this research. Participants were recruited intentionally among people who self-identified with the established profiles. These people had or had had some kind of link with an association or organization that works on gender issues; this link was as an assisted person, worker or collaborator. The audio of these interviews was recorded and, both for its realization and for its recording, signed consent was obtained. Prior to obtaining this consent, the objectives of the research were explained to them, as well as the use that would be made of the results.

The women participants self-identified as people who, in the past, had experienced toxic relationships or hook-ups in which they suffered different types of violence, such as domination, abuse or contempt. At the same time, they identified that, for a time, they remembered those relationships as exciting and explained them to their friends in that way. Today, these women say that they have transformed their memories of these relationships and feel a deep rejection towards them. In the case of men, their narratives, mostly, do not tell us about first-person experiences. They identify situations in their friends’ relationships in which their friends’ partners, having previously experienced toxic relationships, did not treat their friends equally and respectfully. By unequal treatment, they referred to disrespect, disdain and domination.

In all cases, the participants referred to experiences in heterosexual relationships.

### 2.2. Analysis

The discourse of the communicative daily life stories was analyzed according to the following categories: finding flaws in non-dominant partners and criticizing everything they do, belittling non-dominant partners’ abilities, and humiliating and despising them. Initially, the categories were established, taking as a reference the previous scientific literature; later, these categories were revised according to the results obtained, taking as a reference the criterion of transferability. 

In order to establish scientific rigor, following the communicative perspective, in the development of the field work, as well as subsequent dialogues held with the participants, these were always given under pretensions of validity; that is, seeking an understanding and establishing at all times that the arguments were susceptible to criticism. At the same time, once the analysis had been carried out, the results obtained were corroborated by the participants.

### 2.3. Ethical Considerations

The study was conducted according to the guidelines of the Declaration of Helsinki, and approved by the Community of Research on Excellence for All (CREA) ethics committee (protocol code 20210727).

## 3. Results

In the communicative daily life stories with women, different types of treatment of men were identified that may have negative consequences for their health, as they do not correspond to equal treatment. These types were: finding flaws in non-dominant partners and criticizing everything they do, belittling non-dominant partners’ abilities, and humiliating and despising them. These types of treatment were corroborated by the male participants, although, as it has been highlighted in the methodology, they were not always their own experiences but, in the majority of examples, referred to the experiences of close friends.

### 3.1. Finding Flaws in Non-Dominant Partners and Criticizing Everything They Do

The girls of this study who had had previous disdainful hook-ups and had not changed their perception towards them admitted that this behavior had consequences on the way they viewed their later partners. Some of them said that although they defended feminist values, they criticized their non-dominant partners in many aspects, such as finding *micromachismos* or seeing them as more old-fashioned.

In order to understand the following excerpt, it must be said that *micromachismo* (sexist microaggressions) is a concept that refers to the small and invisible sexist or chauvinist attitudes in everyday life, although the concept does not have scientific backing. In Spain, the idea of *micromachismos* has been widely disseminated in some sectors, despite the lack of scientific evidence that proves its link with IPV and the fact that it has not been included as a typology of gender violence [23]. The girls who used this term in our research used it as follows, as Montse explained:

*With sexist boys, we devoted ourselves fully to them, they suppressed us and despised us, with the egalitarian ones we thought about a thousand micromachismos they committed.*

(Montse)

Sofia said that being hooked on a violent boy eliminated any possibility of creating a beautiful relationship with nice boys. She acknowledged that she was stimulated by boys who despised her, and that impeded being motivated by the ones that treated her well.

*This hook on the boy erased everything… The other nice boys that appeared, with whom I had a relationship… It invalidated it… I think because they were really into me… And so I would lose motivation… While I was motivated by the ones who despised me.*

(Sofia)

In the case of Montse, despite recognizing herself as a feminist woman, she revealed that she and her group of friends only hooked up with the most sexist boys, while finding flaws in non-dominant boys. In fact, she said that she even tried to see sexist attitudes in those non-dominant boys, even though she accepted any of the disdainful and sexist attitudes of the violent boys.

*All the girls from my group considered ourselves very feminist and modern, but most of us hooked-up with the ones that looked like ‘machos’, all of them who talked very badly about the women they had hooked up with. Then, the nice ones, at least I, didn’t see them, and I have later realized that I really liked some of them, but I couldn’t permit it because they would crush me [my friends]. So, I ended up crushing them [the nice boys] and stopped seeing them.*

(Montse)

The interview with Daniela reflected the same effect. She found flaws and sexist attitudes in non-dominant boys whilst she submitted herself to boys with violent attitudes towards women.

*In that long period of hook-ups, I saw flaws, even micromachismos, to the most egalitarian boys that treated me the best, that were into me, while I submitted to the sexist boys I hooked up with.*

(Daniela)

In this vein, Juan explained the negative consequences that the concept of *micromachismos* has had on the struggle against gender violence.

*The word micromachismo, apart from having no scientific basis, has been one of the most detrimental words for gender violence. (…) It is a terrible word that has done a lot of harm and has fostered gender violence. It is used as an excuse for those attitudes.*

(Juan)

In addition, Sofia stated that she perceived her boyfriend trying to defend her in front of boys with violent and disdainful attitudes towards her negatively. For example, when one boy tried to kiss her on the mouth without her consent, she saw her boyfriend being annoyed by that wanting to help her as being old-fashioned.

*I remember that there were other boys, especially one that was friends with this other boy… He always tried to kiss me on the mouth… Teasing… Like a ‘progre’ [progressive] thing to do… He would always try it. I remember my boyfriend was so annoyed by that… I didn’t understand much… I thought it annoyed him because he was carca [not modern, old-style] in these things… (…) Now I see he was trying to protect me.*

(Sofia)

Regarding male participants, Juan emphasized that he had seen examples of girls who had had disdainful hook-ups in the past finding flaws in their non-dominant partner in every aspect of everyday life. In his own words, “the thing is to see everything wrong. There is always a flaw, something he does wrong”. In Juan’s interview, a contradiction also appeared between finding sexist attitudes in their non-dominant partners and accepting worse actions in violent boys they have hooked-up with.

*Criticizing the sexism and, however, remembering as the most exciting a boy who has said she was a slut (…) A friend who had a very egalitarian relationship with a very egalitarian guy. They would share the domestic work. (…) One day, she started criticizing his partner a lot, about everything. She said: ‘he seems egalitarian, but he’s not. (…) He takes the shopping list but doesn’t make sure if anything else is missing, which is an incredible inequality’. And I said: ‘Luisa, tell me who you are cheating on him with’. (…) She told me she was cheating on him with a married man from the same group of friends. I said: ‘and the domestic work, the man you are cheating on him with, does he share it too? She said: ‘no, he doesn’t. (…) I don’t care about that, I only have sex with him and that’s it’. So OK, if you don’t care, but, why do you have to look for flaws in your partner if you still consider what the other man does exciting?*

(Juan)

Manuel had seen in the example of his mother’s treatment of his father many attitudes of finding flaws everyday due to his father being in love with her, even when he surprised her.

*My mother had been in toxic relationships previously and because my father was into her she started to see a lot of flaws in him. She saw flaws in everyday things, or did not value nice details that he had. For example, shortly after I was born, my father had to go to the military service, he had to do it far away from home. On one of his leaves, he came to see her by surprise and she, far from being happy, asked him what he was doing there.*

(Manuel)

Lucas also stated that it was common that some women who have been engaged in previous toxic relationships reproduce the role of domination over the more egalitarian men. He also mentioned an apparent contradiction of wanting to dominate but, at the same time, criticizing the lack of initiative of their non-dominant partners.

*In the cases I know, something common that I identify is that they reproduce the roles of inequality. They take on the role of bossy but at the same time the lack of initiative from him. That is to say, they usually criticize any initiative from them: dinners, trips, if they go shopping… But if they stop doing these tasks then they criticize them for lack of initiative. It must also be said that in the end they also end up being leveraged, the relationship loses excitement, there is demotivation, on the part of both.*

(Lucas)

### 3.2. Belittling Non-Dominant Partners’ Abilities 

Some of the participants who remembered a disdainful hook-up as exciting said that they saw their non-dominant partners as lacking knowledge and ability regarding sex. For Violeta, the fact that a non-dominant boy asked her what she liked or not in sex was not exciting and implied he had ‘no idea about sex’.

*I really liked Joan before starting dating him, I knew he was a “good boy”. I liked the first kiss. But nothing more. In the rest of the things I felt like it was evident that he had “no idea about sex” … He asked me “too much” if I wanted this or that thing… I was demotivated completely, I did not feel excited at all. I felt relief when the relationship eventually ended.*

(Violeta)

However, it must be highlighted that some participants such as Daniela admitted not feeling sexual pleasure in the encounters with sexist boys.

*I submitted to the sexist boys I hooked up with even if I didn’t have any pleasure with them.*

(Daniela)

In the interviews with male participants, more examples of belittling non-dominant partners’ abilities appeared. Male participants explained how, throughout their lives, they have seen examples of women who had disdainful past relationships seeing their non-dominant partners as less experienced or worse in sex. In the case of Juan, he stated how double moral standards affect the way these women view their partners regarding sex. As he described, putting violent boys in the ‘fuckzone’ automatically places egalitarian ones in the ‘friendzone’:

*So, if you put him [the one that called you a slut] in the fuckzone, he is for that. So you will have double moral standards and the one who treats you well, you will place him in the friendzone, so he does not know how to have sex, he is not for that. This shatters the sexual relationship with your partner and the boy. Well, if the boy is a simp, it destroys him. If he is an intelligent guy he leaves you and goes with another one who treats him better (…) If you have the fantasy in your head of how the other boy forced you, how you told him ‘no’ but he continued, and you remember it as exciting, how will you get excited with someone that does not force you?*

(Juan)

In the same vein, Lucas also identified other examples of women who had previously had toxic relationships dividing men into two categories: boring or toxic. Thus, non-dominant men were portrayed as dull, while the toxic ones were seen as the exciting ones.

*One of these friends told me: ‘guys are either dull or toxic’. Dull was how they saw guys in an equal relationship.*

(Lucas)

Such attitudes can have very negative consequences for men. David recalls that one of the first girls with whom he had a sexual relationship had been in a toxic relationship.

*The way she treated me gave me a lot of insecurities, especially on a sexual level. I doubted myself for a long time. Over the years, in other relationships where the treatment was completely different, I understood that it was not my problem.*

(David)

This conclusion was also reinforced by Juan. According to him, some conversations among female friends reinforce the idea that non-dominant partners are unattractive or sexually ineffective, which can lead to these men believing this and causing health problems.

But then, you can say it verbally. First, with friends. I’m going to tell you the language they use. ‘he has a smaller d***, the italian guy did have a much bigger d***’. So you are despising him in front of your friends. (…) And they say: ‘his breath stinks sometimes, it looks like he hasn’t brushed his teeth’. So then, the other guy who was at the disco, half drunk and everything, his breath didn’t stink, did it not? (…) It is a constant despise, if it is a nice person he will get a complex about it and will think he doesn’t know like the other guys… It reaches hard situations like depression, and even suicide.

(Juan)

At the same time, there is a consensus that dissatisfaction in relationships has serious health effects. In this regard, David recounted the example of a friend who experienced a relationship in which she, after having lived in a toxic relationship, entered into a relationship with him. In this relationship, she found stability at all levels, but she did not have a passionate relationship with him and even ended up treating him like a sick person. All this made his friend fall ill, and he only overcame his health problems when he ended this relationship and started a completely different one.

*She did not see him as an attractive person, a person she was attracted to, simply as someone who could give her a good life on the level of security and a way out of the situation she had in her previous history. He started to feel bad, there were even periods when he was on sick leave, he was constantly anxious… He was on sick leave for long periods of time because of anxiety. He felt unwell and out of place in the world. He only came out of it the moment he left this relationship and found a relationship with meaning, attraction and love.*

(David)

### 3.3. Humiliating and Despising

Humiliation and despisal towards the non-dominant partners of the girls who still remember a violent relationship as exciting are also evident in the interviews. These attitudes include ignoring and flirting with other boys with violent attitudes in front of the non-dominant boys. Daniela reported ignoring and confusing the nice guys she knew while she was subjected to boys who despised her.

*I have always treated very badly and despised all the nice boys I have been with. For example, I ignored two of them after hooking up. I treated one very badly, telling him yes and no all the time so he came after me, but knowing I didn’t like him. The other one, we dated for two weeks and after telling him it was better to leave it, I told him I felt really relieved of having broken up with him.*

(Daniela)

Juan mentioned that having a good memory about a past violent hook-up can lead to cheating on non-dominant partners. As he explained, in the same way that the most jealous boys are the ones who cheat the most, the girls who remember a violent hook-up or relationship as exciting are the ones who cheat the most and who get angry if their egalitarian boyfriend leaves them, even without cheating.

*Those girls who remember that as exciting are the ones who cheat the most, and they do it with the worst guys, the ones who despise them the most (…) Those girls are the same as the boys that have double moral standards. If the boys, without cheating on them, simply say: ‘well, look, I want to end the relationship.’ They call him every name in the book!*

(Juan)

Cheating and flirting with boys with violent attitudes appeared in the girls’ interviews. Violeta explained that she cheated on a boy who was into her. She cheated on her boyfriend or fooled around with other boys in order to feel above her partner.

*When I was 16 I hooked up with Fernando in the disco, but he was an acquaintance of the neighborhood… he did not have bad vibes. He had a boyfriend-like attitude toward me. He came to pick me up at the high school, he treated me very well. On the one hand, I was happy because “finally someone wanted to be my boyfriend” but at the same time, I thought ‘I’m going to get bored with this boy’. Even if I found him objectively handsome, I always saw many flaws in him. (…) One day, I decided to cheat on him, so it was “left clear” that I would not only be with him (…) it was for me to feel good humiliating him that way. Another day, we went to the cinema, with a friend of his and his girlfriend. The friend was a flirt… I liked him better. (…) I was flirting with him. I think it was very humiliating for my boyfriend (…) after that day, he changed.*

(Violeta)

Juan explains the humiliation as something closely linked to a false memory of a violent relationship.

*It is a constant need to humiliate. It is a desire to humiliate. As she hasn’t had sexual satisfaction in what she remembers as so exciting, she needs another excitation, the humiliation, despise and deceit. To feel like they are feeling any emotion at all.*

(Juan)

Sometimes this humiliation not only implies domination by her towards him, but is also exercised by men who respond to traditional models of masculinity. As Juan explained, the violent boys who had had disdainful hook-ups with the women like to remind their actual partners what they did, and sometimes, the woman does not stand against them.

*In a dinner with parents of a school, a man said three times to another man, and everyone could hear it, including the women. He said: ‘I knew your wife before you’. Three times he said it. It was clear what that meant. The woman does not defend his husband, she even finds it funny what the other one is saying’.*

(Juan)

## 4. Discussion

The results reveal that female participants who had maintained a false memory of previous toxic relationships reproduced attitudes of disdain in their later egalitarian relationships with non-dominant men. Male participants also indicated that women who had previous toxic relationships or hook-ups tended to ill-treat men who treated them well. These attitudes include finding flaws in non-dominant partners while forgiving the sexist and violent attitudes of boys with a traditional dominant masculinity; belittling non-dominant partners’ abilities, especially regarding sex; and humiliating non-dominant partners by confusing them on purpose, ignoring them and cheating and flirting with men with a traditional dominant masculinity. 

Intimate partner violence is defined as the physical, emotional, sexual or psychological abuse or violence committed by a partner or acquaintances [24]. Thus, the findings of this study are in line with the definition and the previous scientific literature that has shown that both men and women can be either victims or perpetrators of such violence [1,13]. Although the health consequences that IPV has on women have been widely researched [2,5,6,25], the findings of this study support the need for further into the study health consequences of IPV on non-dominant men in future relationships [1]. 

There is general knowledge about the consequences of some of the attitudes presented here. More specifically, the scientific literature has evidenced how humiliation is a very intense negative emotion that stays in an individual’s memory for longer than other negative emotions, especially when there is an audience who laughs at such an attitude of despisal [26]. Infidelity to the non-dominant partners was also demonstrated in the interviews. The scientific literature has emphasized how infidelity is often followed by intense feelings of horror, despair, anguish and sadness, among others [27], and it is associated with depressive symptoms [28]. 

However, the participants in this study demonstrated a difference regarding masculinities’ response towards the attitudes mentioned. Whereas those who respond to a traditional oppressed masculinity have been reported to suffer these consequences more often, men who show attitudes of a new alternative masculinity do not tolerate them and leave these relationships. This finding is consistent with recent research that has pointed out new alternative masculinities stand against double moral standards [29] and humiliation or despisal towards them [30].

Another element appearing in the interviews is related to the double moral standards of those female participants who had not recovered from the trauma of a past violent hook-up or relationship. In this vein, the classic double sexual standard that has historically judged women more severely than men for having a similar sexual behavior, even in what are considered liberal hook-up contexts [31], is reproduced in reverse. Historically, this patriarchal double morality has differentiated two types of women: the slut, and the sexless wife [32]. However, some participants in the study reported double moral standards for classifying men into two categories: the *friendzone* and the *fuckzone.* This finding is consistent with the previous literature that evidenced how women who had hook-ups with violent boys and remembered them as exciting and sexy viewed their relationship with an non-dominant boyfriend as based on routine and comfort, but not passion [11]. In essence, the classic patriarchal double moral standards are reproduced [33], where desire is linked to men with power but no ethical values, and friendship to non-dominant men [32]. These findings should be taken into account in order to achieve the goals of our society, such as gender equality, which is essential for the progress of nations [34]. However, if egalitarian men are regarded as boring and aggressive ones are categorized as attractive, the achievement of this goal is blocked [32]. For both men and women who are victims of this double standard, this generates dissatisfaction in their relationships, which, as one of the stories showed us, can lead to serious health problems. However, little is known about the kind of illnesses they can develop and how to deal with them.

On the other hand, the use of the word *micromachismo* has been identified by participants, both male and female, as an excuse to criticize non-dominant men while forgiving the most sexist and chauvinist attitudes of other men. The concept of *micromachismo* refers to small sexist attitudes that are often not identified and includes colour distinction for males and females and giving the check in a restaurant to the man, among other attitudes. Through the use of this concept, according to participants, those attitudes of non-dominant men which do not include violence are categorized as sexist, while men who perpetrate violence against women do not receive such criticism. This is in line with previous studies that pointed out this kind of criticism towards men with an oppressed masculinity but not towards men with violent features [35]. Related to the theory of *micromachismo*, the participants in this study explained how it implies that all men are the same, while the scientific literature has shown that this is not so [36,37].

Taking into account the previous scientific literature mentioned in this discussion, more specifically the work of Racionero [9,38,39], memory plays an important role in the reproduction or overcoming of IPV. The application of this theory to our results provides us with the main starting point for carrying out preventive work. This seeks to work on the memories of relationships that have led to the socialization of violent relationships [10,14].

Our results also point to the importance of working with men to help them to identify these types of situations and not accept this treatment. In this sense, scientific research has also shown how men with a new alternative masculinity [22] do not accept double standards and help to overcome the pattern in which some women reproduce the behavior of traditional dominant masculinities [26,29] and only want egalitarian and passionate relationships [34].

The limitations of the current study must be addressed. Many respondents have talked about other people’s experiences to illustrate the issue. In addition, only heterosexual relationships have been taken into account. Future works should extend the research to men who have been victims of such treatment, since in our research, in general, they talk about the experiences of friends. This would make it possible to go deeper into the health consequences for these men. In the case of women, they themselves have highlighted the contradiction of defending feminist values but not putting them into practice in their personal lives. It would be interesting to delve into the causes of this contradiction and the consequences it may have on the development of this social movement.

## 5. Conclusions

Our research shows that previous violent relationships, if they are not adequately analyzed and worked through in the recovery process, also have consequences in subsequent relationships of participants of the study, even if they are egalitarian. Those women participants who had not overcome the false memory created about past relationships or hook-ups tended to treat their later non-dominant partners badly. However, at the same time, we have also found it demonstrated that, when the analysis on the false memory is performed and the memory is changed, women participants who have had toxic hook-ups and relationships can decide to have egalitarian and passionate relationships.

These results are very useful for advancing interventions in gender-based violence. On the one hand, they point out that, after experiencing gender-based violence, the choice of non-dominant partners is not sufficient to ensure satisfactory relationships free of violence. When the above-mentioned changes have not taken place, non-dominant men may be treated in a way that has serious consequences for their health. In turn, these findings provide a rigorous, scientific explanation for the dissatisfaction that sometimes occurs in supposedly egalitarian relationships. They also allow us to identify one of the possible causes of health problems in men, putting the focus for the first time on gender-based violence previously suffered by their partners. The extent of the shadow of violence, and of its consequences, is wider than has been identified and understood so far.

Therefore, the intervention should focus on working on the victims’ memories of their experiences, removing any possible link with excitation. In the case of intervention with men, these results reinforce the importance of not blaming all men for the existence of gender-based violence. Those responsible are those who respond to the traditional dominant model of masculinity and perpetrate this violence. When working on the construction of a masculine identity which corresponds to the new alternative masculinity model, the identification and non-acceptance of any mistreatment, also on the part of women who have previously been victims, must be deepened. Such mistreatment can not only damage self-esteem, but also have other serious effects on their health and, as in the case of women, on future relationships.

## Data Availability

The data presented in this study are available on request from the corresponding author. The data are not publicly available due to ethical restrictions.
